# Etoposide Induces Apoptosis in Activated Human Hepatic Stellate Cells via ER Stress

**DOI:** 10.1038/srep34330

**Published:** 2016-09-29

**Authors:** Chen Wang, Feng Zhang, Yu Cao, Mingming Zhang, Aixiu Wang, Mingcui Xu, Min Su, Ming Zhang, Yuzheng Zhuge

**Affiliations:** 1 Department of Gastroenterology, Drum Tower Hospital, Medical School of Nanjing University, Nanjing, China; 2 Department of Gastroenterology, Affiliated Drum Tower Clinical Medical School of Nanjing Medical University, Nanjing, China

## Abstract

The activation of hepatic stellate cells (HSCs) plays a vital role in the progression of liver fibrosis, and the induction of HSCs apoptosis may attenuate or reverse fibrogenesis. The therapeutic effects of etoposide(VP-16), a widely used anticancer agent, on HSCs apoptosis and liver fibrosis resolution are still unclear. Here, we report that VP-16 reduced the proliferation of LX-2 cells and led to significantly high levels of apoptosis, as indicated by Annexin V staining and the proteolytic cleavage of the executioner caspase-3 and PARP. Additionally, the unfolded protein response regulators CHOP, BIP, caspase-12, p-eIF2α and IRE1α, which are considered endoplasmic reticulum (ER) stress markers, were upregulated by VP-16. The strong inhibitory effect of VP-16 on LX-2 cells was mainly dependent on ER stress, which activated JNK signaling pathway. Remarkably, VP-16 treatment decreased the expression of α-SMA and type I collagen and simultaneously increased the ratio of matrix metalloproteinases (MMPs) to tissue inhibitor of matrix metalloproteinases (TIMPs). In contrast, VP-16 induced significantly more apoptosis in HSCs than in normal hepatocytes. Taken together, our findings demonstrate that VP-16 exerts a proapoptotic effect on LX-2 cells and has an antifibrogenic effect on collagen deposition, suggesting a new strategy for the treatment of liver fibrosis.

Liver fibrosis is a reversible wound-healing response to all etiologies of liver disease, including chronic viral hepatitis, alcohol consumption, fatty liver disease, cholestasis and autoimmune hepatitis^1^. Sustained fibrogenesis leads to cirrhosis, and even primary liver cancer. A key discovery in understanding fibrosis was that hepatic stellate cells (HSCs) are the primary effector cell in the liver. In the normal liver, HSCs are quiescent and nonproliferative, and they reside in the space of Disse. Their main function is to store vitamin A and other retinoids. Upon liver injury, HSCs proliferate and transform into a myofibroblast-like phenotype; they express α-smooth muscle actin (α-SMA) and secrete excess extracellular matrix (ECM), which is predominantly type I collagen^2^. HSCs also produce a tissue inhibitor of matrix metalloproteinases (TIMPs), thus shifting the balance of the ECM towards the deposition of collagen and contributing to fibrosis^3^. Because HSCs play a critical role in liver fibrosis, the resolution of fibrosis involves pathways that cause either HSCs apoptosis, senescence, or reversion to their quiescent stage^2^. Apoptosis is an essential mechanism for cell clearance. In recent years, antifibrotic drug research has focused on the promotion of apoptosis in activated HSCs; indeed, the induction of HSCs apoptosis by gliotoxin, proteasome inhibition or sorafenib reduces liver fibrosis^4^,^5^,^6^. Thus, the induction of apoptosis in activated HSCs during the recovery phase of liver fibrosis represents a therapeutic antifibrogenic strategy.

Etoposide(VP-16) is one of the most widely used cancer chemotherapy agents for improved treatment of a variety of human malignant tumors, including primary liver cancer^7^,^8^,^9^. Previous studies have indicated that VP-16 activity is mediated by its interaction with topoisomerase II, which leads to DNA strand breaks that are lethal to the cell^10^. Recent reports have demonstrated that VP-16 may possess an uncharacterized cytotoxic function via other channels, such as the induction of reactive oxygen species (ROS), the stimulation of Akt/mTOR signaling, and the activation of caspase-3^11^,^12^,^13^. Additionally, the action of VP-16 can be either p53-dependent or p53-independent^14^,^15^. The mechanism by which VP-16 initiates the intracellular apoptotic pathway is not well understood; nevertheless, it may vary from cell type to cell type. However, it is unknown whether VP-16 affect the apoptosis of HSCs.

The purpose of the present study was to evaluate the influence of VP-16 treatment on liver fibrosis in the LX-2 cell line by investigating the effects of VP-16 on the proliferation, apoptosis and collagen expression of HSCs.

## Results

### VP-16 inhibits the proliferation of HSCs and induces G_2_/M cell cycle arrest

**Figure 1:**
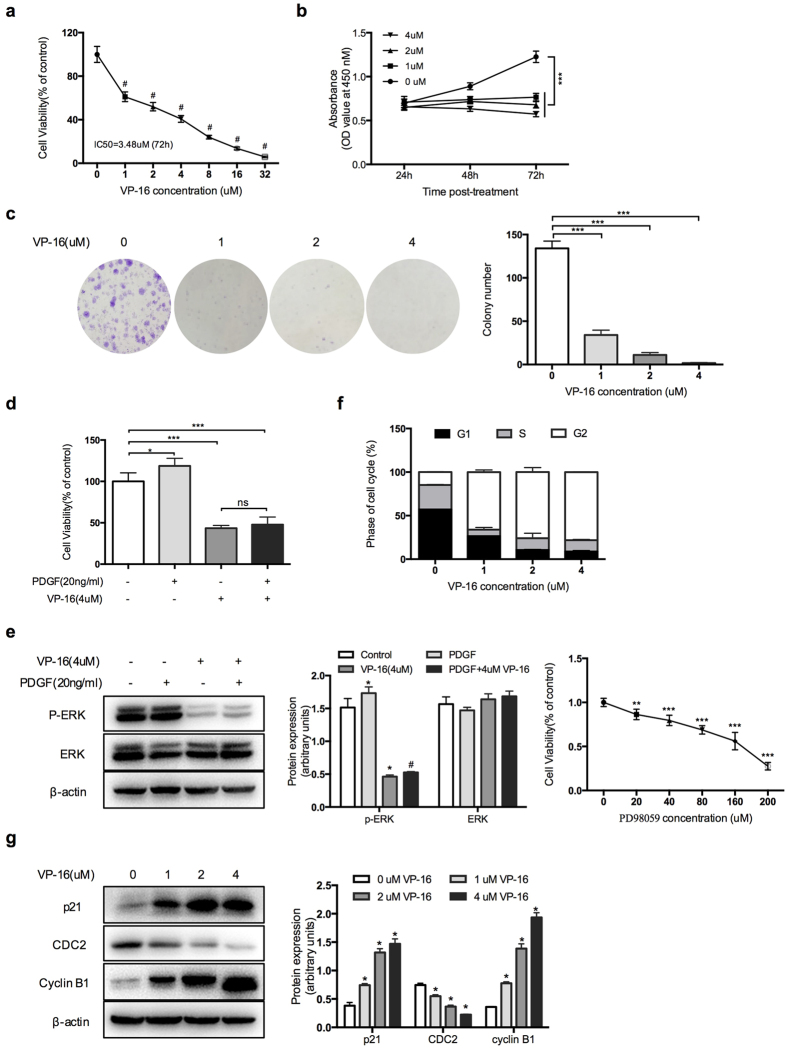
VP-16 inhibits LX-2 cells proliferation and induces G_2_/M cell cycle arrest. (**a**) The cytotoxicity of VP-16 was evaluated in activated human LX-2 hepatic stellate cells. LX-2 cells were treated with vehicle (0.1% DMSO) or various concentrations of VP-16 for the indicated times. The cytotoxicity was assessed using the CCK-8 assay. The results are expressed as the mean ± SD of three independent experiments. ^#^P < 0.0001, compared with the control group. (**b**) LX-2 cells were treated with 0–4 μM VP-16 over a course of 72 h, and the relative absorbance at 450 nM was analyzed to probe the time dependence of the VP-16 effect. (**c**) A representative experiment of the colony-formation assay of VP-16-treated cells. LX-2 cells were grown for 14 days, treated with 0–4 μM of VP-16, and stained with 0.5% crystal violet. The data shown here are from three independent experiments with similar results. (**d**) LX-2 cells were stimulated with VP-16 (4 μM) for 72 h with or without pretreatment with platelet-derived growth factor (PDGF; 20 ng/mL) for 1 h. The cell viability was assessed using the CCK-8 assay. (**e**) Left: LX-2 cells were treated as described above. The activation of extracellular signal-regulated kinase (ERK) was assessed by western blotting analysis for phosphor-ERK. Right: LX-2 cells were treated with vehicle (0.1% DMSO) or various concentrations of PD98059 for 72 h. Cell viability was assessed using the CCK-8 assay. ^*^P < 0.05, compared with the control group. ^#^P < 0.05, compared with the group treated with PDGF alone. (**f, g**) LX-2 cells were treated with 0–4 μM VP-16 for 72 h. Cell cycle analysis was performed using flow cytometry, and the levels of cell cycle-regulating proteins were analyzed by western blotting. β-actin was used as a loading control. The results are expressed as the mean ± SD of three independent experiments. *P < 0.05, **P < 0.01, ***P < 0.001.

LX-2 cells were incubated for 72 h in medium with VP-16 at a range of concentrations (0, 1, 2, 4, 8, 16, and 32 μM), and the cell viability was evaluated using the CCK-8 assay. As shown in Fig. 1a, the cell viability was significantly reduced by VP-16 in a dose-dependent manner, and the IC_50_ value was 3.48 μM, which is much lower than the values reported for various types of tumor cells^16^,^17^. Next, we treated LX-2 cells with low-dose VP-16 over a course of 72 h. The CCK-8 assay analysis showed the time-dependent anti-proliferation effect of VP-16 (Fig. 1b). Furthermore, VP-16 treatment almost completely inhibited the formation of LX-2 cells colonies, as determined by colony formation assays (Fig. 1c). This inhibitory effect remained pronounced compared with those observed after pre-stimulation by platelet-derived growth factor (PDGF; Fig. 1d). Western blotting analysis showed that the stimulation of LX-2 cells with PDGF induced extracellular signal-regulated kinase (ERK) activation, which could be inhibited by VP-16 (Fig. 1e). Moreover, the proliferation of LX-2 cells was inhibited by PD98059, an ERK inhibitor, which inhibits the MAPK signaling pathway (Fig. 1e). To further study the inhibitory effect of VP-16 on cell growth, we investigated the cell cycle distribution by flow cytometry. Cell cycle analysis showed a marked increase in the number of G2 phase cells and a decrease in the number of G1 and S phase cells after VP-16 treatment at a higher concentration of VP-16 compared with the control (Fig. 1f and S1a). Concordantly, the expression levels of cyclin B1 and p21, which are known to regulate the G_2_/M phase transition, were markedly increased, and the CDC2 level was reduced (Fig. 1g).

### VP-16 induces HSCs apoptosis via the caspase-dependent mitochondrial apoptotic pathway

**Figure 2:**
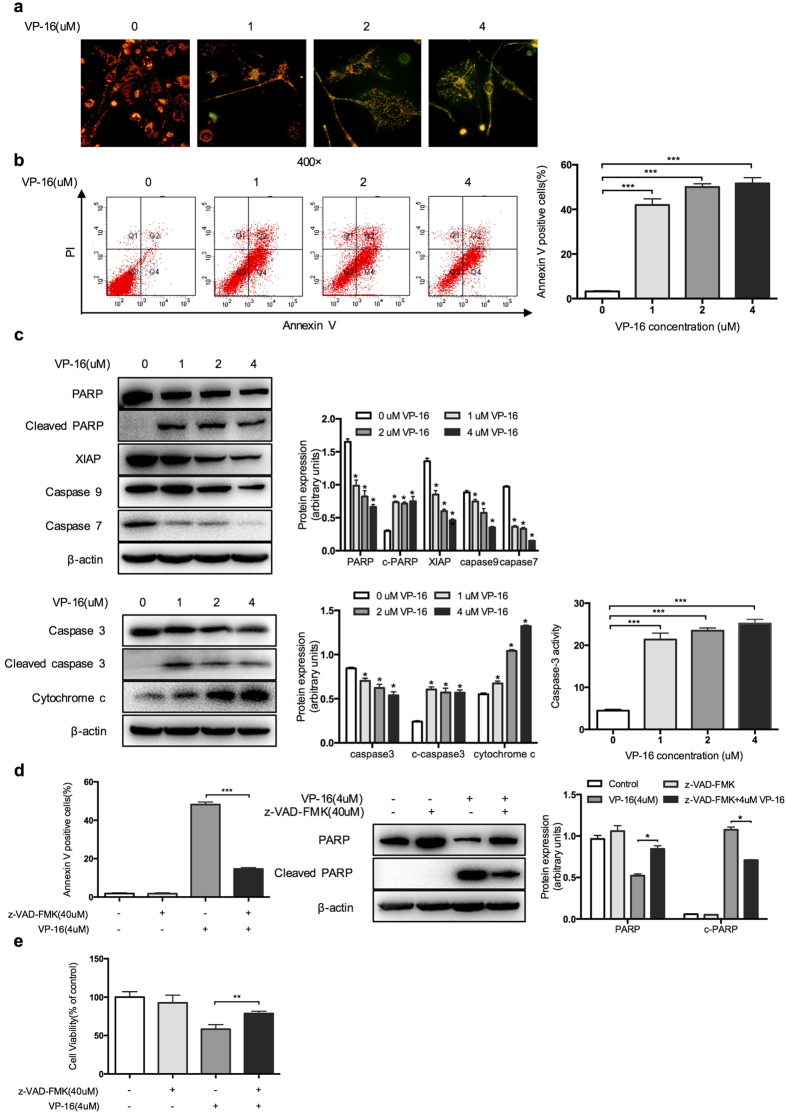
Effect of VP-16 on the induction of apoptosis in LX-2 cells. (**a**) LX-2 cells were stained with JC-1 after incubation with VP-16 for 72 h to determine ΔΨm (magnification × 400). Undamaged mitochondria are hyperpolarized and stained red by the JC-1 dye. When mitochondria are damaged by VP-16, ΔΨm decreases, and the JC-1 dye diffuses out of the mitochondria. Thus, the dye shows green fluorescence. (**b**) LX-2 cells were treated with various concentrations of VP-16 for 72 h. Apoptotic cells were analyzed using flow cytometry. The percentage of Annexin V-positive cells relative to untreated controls is indicated in a bar chart. (**c**) LX-2 cells were treated as described above. Western blotting was used to analyze the expression of PARP, cleaved PARP, XIAP, caspase-9, caspase-7, cytochrome *c*, caspase-3 and cleaved caspase-3. β-actin was used as a loading control. VP-16 increased the caspase-3 activities of LX-2 cells, which was assessed using a Caspase-3 Activity Assay kit. (**d**,**e**) LX-2 cells were pretreated with z-VAD-FMK (40 μM) for 1 h and then with 4 μM VP-16 for 72 h. (**d**) Annexin V-positive cells were examined by flow cytometry. PARP and cleaved PARP were measured by western blotting. β-actin was used as the loading control. (**e**) Cell viability was assessed using the CCK-8 assay. The results are expressed as the mean ± SD of three independent experiments. *P < 0.05, **P < 0.01, ***P < 0.001.

After exposure to VP-16, LX-2 cells shrank, aggregated, or even floated, suggesting that apoptotic events may occur. JC-1 fluorescence was used to detect the mitochondrial depolarization that occurs in the early stages of apoptosis. Apoptotic cells were identified based on a decrease in red fluorescence and an increase in green fluorescence in the cytoplasm. The increased proportions of green-stained cells suggested a strong proapoptotic effect of VP-16 on LX-2 cells (Fig. 2a). Next, we stained LX-2 cells with Annexin V-FITC and PI. As shown in Fig. 2b, the proportion of Annexin V-positive cells, which are considered apoptotic cells, was significantly increased. Consistently, the levels of PARP, XIAP, cytochrome c and caspases, which are hallmarks of apoptosis, were reduced by VP-16 in a dose-dependent manner. Strikingly, the treatment with VP-16 significantly increased the expression of cleaved caspase-3 and the activity of caspase-3 in LX-2 cells (Fig. 2c). Caspases are a family of cysteine proteases that are essential to the apoptotic pathway. To further demonstrate the involvement of caspases activity in the apoptosis induced by VP-16, LX-2 cells were cultured with 4 μM VP-16 in the absence or presence of a pan-caspase inhibitor (z-VAD-FMK, 40 μM). Flow cytometry analysis showed that the percentage of apoptotic cells in the z-VAD-FMK-pretreated group was lower than that in the group treated with VP-16 alone. Western blotting showed that the activation of PARP cleavage triggered by VP-16 was abrogated by z-VAD-FMK (Fig. 2d). Subsequently, a CCK-8 assay demonstrated that pretreatment with z-VAD-FMK abrogated the anti-proliferation effect induced by VP-16 (Fig. 2e).

### VP-16 inhibits collagen synthesis and induces collagen degradation in HSCs

**Figure 3:**
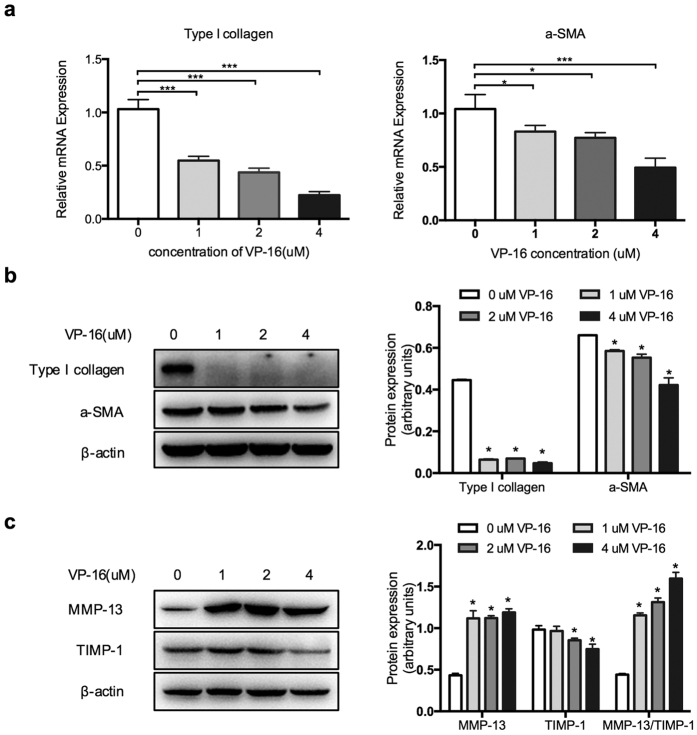
VP-16 reduces collagen deposition in LX-2 cells. (**a**–**c**) LX-2 cells were treated with various concentrations of VP-16 for 72 h. (**a**) The mRNA expression levels of type I collagen and α-SMA were determined using real-time PCR. (**b**) The protein levels of type I collagen and α-SMA were measured by western blotting. β-actin was used as a loading control. (**c**) VP-16 regulates the expressions of TIMP-1 and MMP-13, as shown by western blotting analysis. β-actin was used as a loading control. The data are presented as the mean ± SD of three independent experiments performed in triplicates. *P < 0.05 ***P < 0.001.

Next, we probed fibrotic indices, including the myofibroblast marker α-SMA and type I collagen, at the mRNA and protein levels using real-time PCR and western blotting analysis, respectively. Interestingly, VP-16 significantly reduced the mRNA levels of intracellular type I collagen and α-SMA compared to the levels observed in LX-2 cells treated with DMSO (Fig. 3a). Concordantly, western blotting showed that exposure to VP-16 resulted in markedly decreased expression of both α-SMA and type I collagen (Fig. 3b). The outcome of fibrosis is influenced by the synthesis of collagen and the degradation mediated by matrix metalloproteinases (MMPs). The actual proteolytic activity of MMPs depends on the ratio of MMPs to their corresponding TIMPs. The balance between MMPs and TIMPs regulates ECM turnover and remodeling during normal development and pathogenesis^18^,^19^. Here, we tested whether treatment with VP-16 contributes to MMPs regulation in fibrosis. As shown in Fig. 3c, western blotting analysis suggested that the increased TIMP-1 expression was inhibited by VP-16 when MMP-13 was activated. Thus, the ratio of MMP-13 to TIMP-1 was substantially increased, indicating that the MMP/TIMP balance changed in favor of fiber degradation.

### VP-16 induces the activation of the ER stress pathway

**Figure 4:**
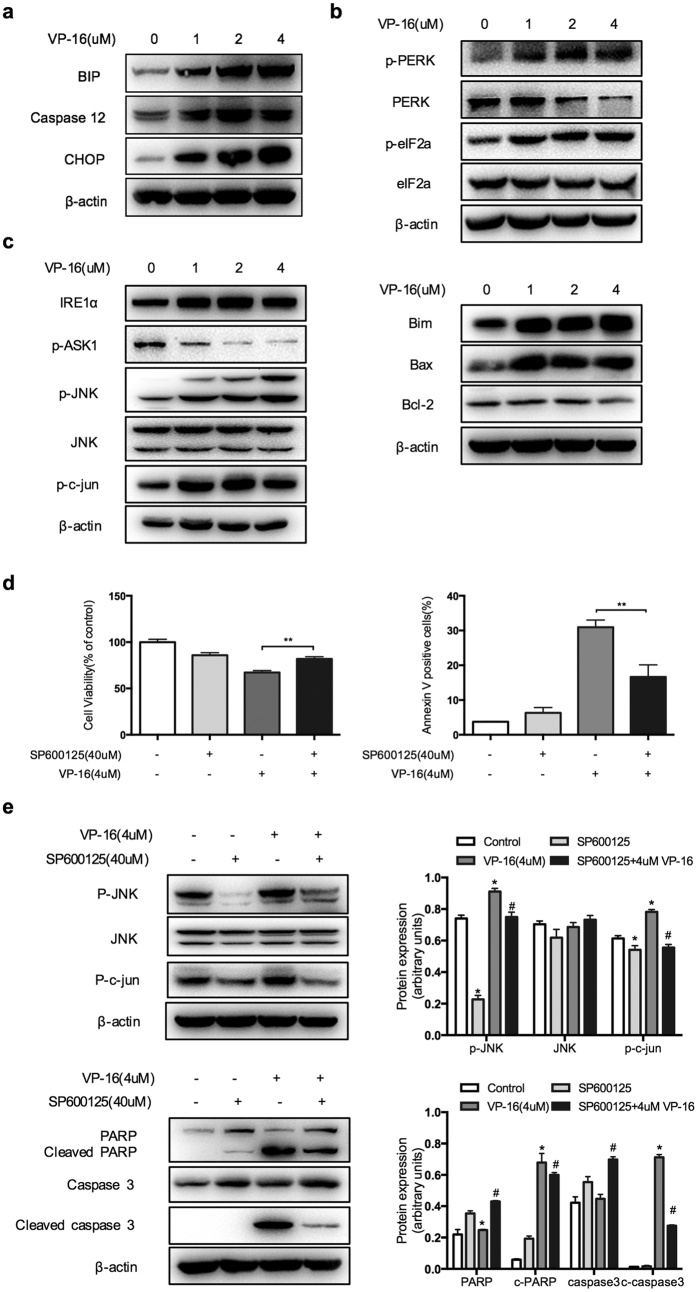
Activation of the ER stress signaling pathway leads to the LX-2 cell apoptosis induced by VP-16. (**a**) Western blotting analysis of ER stress-associated proteins in LX-2 cells after treatment with VP-16 for 72 h. (**b**) After exposure to VP-16 for 72 h, the protein phosphorylation levels of PERK and eIF2α were analyzed by western blotting. (**c**) After exposure to VP-16 for 72 h, the proteins of the IRE1α /ASK1/JNK signaling pathway were analyzed by western blotting. (**d**,**e**) LX-2 cells were pretreated with a JNK inhibitor (SP600125, 40 μM) for 1 h, and then treated with 4 μM VP-16 for 72 h. (**d**) Cell viability was measured using the CCK-8 assay. The percentage of apoptosis cells was analyzed by flow cytometry. The data are presented as the mean ± SD of at least three independent experiments performed in triplicates. **P < 0.01, compared with the group treated with VP-16 alone. (**e**) Western blotting analysis of the cell lysates was performed using the indicated antibodies. In all of the western blotting analyses, β-actin was used as a loading control. *P < 0.05, compared with the control group. ^#^P < 0.05, compared with the group treated with VP-16 alone.

Endoplasmic reticulum (ER) stress is considered a major cause of apoptosis in many biological processes. We speculated that VP-16 might induce ER overload and ER stress, resulting in the unfolded protein response (UPR)-triggered apoptosis of HSCs. As expected, VP-16 treatment resulted in the upregulation of the protein levels of BIP, caspase-12 and CHOP, which are considered general ER stress biomarkers (Fig. 4a). Furthermore, the protein phosphorylation levels of PERK and eIF2α, which form one of the UPR signaling branches, were significantly increased after treatment with VP-16 (Fig. 4b). To further investigate the relationship between the activation of PERK/ eIF2α and apoptosis, LX-2 cells were pretreated with salubrinal, a selective phosphatase inhibitor of eIF2α. However, pretreatment with salubrinal failed to reduce the resulting apoptosis (Fig. S1b). We found that alterations in another UPR signaling branch, the IRE1α branch, which promotes apoptosis in response to ER stress, resulted in the activation of apoptosis signal-regulating kinase 1 (ASK1) through the dephosphorylation of Ser967. Activated ASK1 led to the phosphorylation of c-Jun N-terminal kinase (JNK), which further activated the proapoptotic cytokines Bim and Bax and downregulated the antiapoptotic cytokine Bcl-2 (Fig. 4c). In ER stress-induced cellular apoptosis, the mitochondrion plays a secondary role, as indicated by the upregulation of cytochrome c and cleaved caspase-3 (Fig. 2c). Moreover, VP-16 also upregulated the protein phosphorylation levels of p38 MAPK (Fig. S2a). To support the finding that VP-16-induced apoptosis is mediated by the JNK signaling pathway, we incubated cells with SP600125, a JNK inhibitor. As shown in Fig. 4d, pretreatment of LX-2 cells with SP600125 restored cell viability to a near maximal level by reducing the resulting apoptosis. Consistently, western blotting analysis showed that p-JNK and the downstream protein c-jun were downregulated and that PARP/caspase-3 activation was inhibited significantly in cells pretreated with SP600125 (Fig. 4e). These results indicated that VP-16 triggers apoptosis through the ER stress-mediated activation of the IRE1/ASK1/JNK pathway.

### VP-16 exerts more potent effect on activated HSCs than on normal hepatocytes

**Figure 5:**
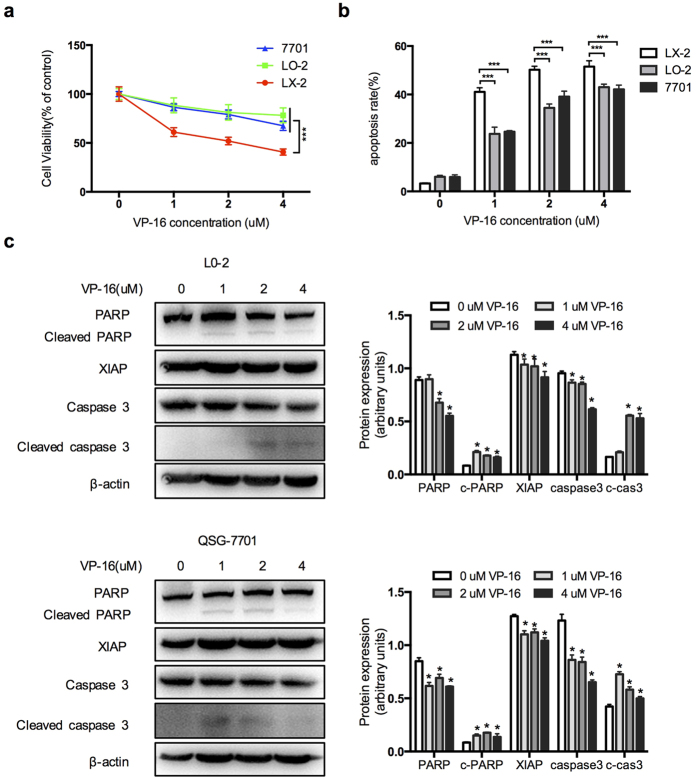
VP-16 had a more potent effect on LX-2 cells than normal hepatocytes. (**a**–**c**) Three cell lines were treated with 0–4 μM VP-16 for 72 h. (**a**) Cytotoxicity was measured using the CCK-8 assay. (**b**) Apoptotic cells were analyzed using flow cytometry. Annexin V-positive cells are displayed in the bar chart as a percentage of the untreated controls. (**c**) Apoptosis-regulating protein levels were analyzed by western blotting. β-actin was used as a loading control. The data are presented as the mean ± SD of three independent experiments. *P < 0.05, ***P < 0.001.

*In vivo*, an ideal antifibrogenic drug should be cytotoxic to HSCs while having limited side effects on normal cells. To explore the effect of VP-16 on normal hepatocytes, we used two cell lines, LO-2 and QSG-7701, treated with the same concentration of VP-16 as for the LX-2 cells. As shown in Fig. 5a, VP-16 caused significantly more cell death in LX-2 cells than in LO-2 and QSG-7701 cells. Consistently, the percentage of cells exhibiting apoptosis was higher for LX-2 cells than for LO-2 and QSG-7701 cells at the same VP-16 concentration (Fig. 5b). Western blotting analysis showed that the levels of apoptosis-regulating proteins changed slightly in these cells compared to LX-2 cells (Fig. 5c). These results indicated that VP-16 had a more potent effect on activated HSCs than on normal hepatocytes.

## Discussion

Liver fibrosis can be reversed through the apoptosis of activated HSCs^20^. The use of VP-16 has been recommended as an important chemotherapeutic agent to treat a wide spectrum of human cancers. Here, we firstly established that VP-16 has a pivotal role in the induction of HSCs apoptosis, which may open a new opportunity for therapeutic strategies to reverse liver fibrosis. Five significant findings are presented in this study: (1) VP-16 inhibited the proliferation of activated HSCs and caused apoptosis in activated HSCs in a caspase-dependent manner; (2) VP-16 mediated a reduction in collagen deposition in activated HSCs; (3) the VP-16-induced apoptosis mainly depended on ER stress; (4) VP-16 treatment activated multiple molecular mechanisms involving caspase-12, CHOP, JNK and Bcl-2/Bax for apoptosis; and (5) JNK was essential for VP-16-induced apoptosis.

Apoptosis or programmed cell death is a key regulator of physiological growth control and the regulation of tissue homeostasis. Many reports have demonstrated that VP-16 inhibits the proliferative activity of various tumor cells and induces their apoptosis^21^. In the present study, VP-16 was capable of inhibiting activated human LX-2 hepatic stellate cell proliferation in a dose- and time-dependent manner. Moreover, the inhibitory effect remained pronounced with the removal of VP-16 (Fig. S2b). PDGF is the most potent mitogen of HSCs and stimulates ERK, which is a key molecule in the signaling pathways of the proliferation of HSCs during liver fibrogenesis^22^. We found that blocking ERK inhibited the proliferation of LX-2 cells and that VP-16 inhibited PDGF-induced ERK activation. Thus, we speculate that VP-16 inhibits the proliferation of HSCs by inhibiting the phosphorylation of ERK *in vitro*. We also found that upon VP-16 induction, cell death began with growth arrest, as indicated by the accumulation of cells in the G_2_ phase of the cell cycle. These effects were similar to those previously reported in human non-small cell lung cancer cells and mouse embryo fibroblasts (MEFs)^23^,^24^. These cells usually respond to genotoxic stress by arresting at the G_2_/M transition and then undergoing programmed cell death^25^. Consequently, we evaluated mitochondrial depolarization which occurs in the early stages of apoptosis. JC-1 fluorescence suggested the occurrence of apoptosis, and Annexin V/PI staining further confirmed that VP-16 induced apoptosis in a dose-dependent manner. Mitochondrial depolarization, which is considered an irreversible step in the apoptotic process, can trigger a cascade of caspases. Our study also strongly indicated that VP-16-induced apoptosis occurred in a caspase-dependent manner, as established through experiments using a caspases inhibitor.

The ER plays a major role in the synthesis, folding, and structural maturation of more than a third of all proteins made in the cell^26^. ER stress is brought on by the accumulation of misfolded proteins in the ER, which triggers an adaptive program called the UPR. The UPR is a conserved pathway that transmits signals to restore homeostasis or eliminate irreparably damaged cells^27^. After VP-16 exposure, PERK and eIF2a are phosphorylated, and the expression of ER-resident chaperones, such as BIP and caspase-12, was also upregulated. These signaling transduction pathways are adaptation responses to prevent the further accumulation of misfolded proteins. If ER stress is persistent or excessive, the failure to restore ER functions results in cell death, typically apoptosis. We found that VP-16 also activated CHOP, a key transcription factor induced by ER stress whose overexpression can lead to growth arrest and apoptosis^28^. The IRE1 branch governs the most conserved UPR signaling pathway, which has acquired additional functions that are thought to promote apoptosis in response to ER stress^29^. One proposed proapoptotic output of IRE1 signaling may be its activation of JNK^30^. Previously, studies have reported that both JNK and CHOP eliminate the anti-apoptotic effect of Bcl-2; CHOP blocks the expression of Bcl-2, whereas JNK phosphorylates Bcl-2. JNK also phosphorylates Bim, which leads to its release from the cytoskeleton and induces Bax-dependent apoptosis^31^,^32^. In our experiments, treatment with VP-16 activated IRE1α/ASK1/JNK and triggered the Bcl-2 family proteins. To obtain further data supporting the idea that VP-16-induced apoptosis is mediated through the IRE1α/ASK1/JNK pathway, we incubated cells with SP600125, a JNK inhibitor. In LX-2 cells, SP600125 effectively attenuated VP-16-induced apoptosis, as shown by Annexin V staining and the CCK-8 assay, resulting in diminished concentrations of the apoptotic marker protein cleaved PARP and caspase-3. Thus, these findings strongly indicate that ER stress is a crucial target for triggering cell apoptosis in activated HSCs.

Importantly, we have demonstrated that the proapoptotic effect of VP-16 has greater specificity for activated HSCs than normal hepatocytes. During liver fibrosis, the apoptosis of hepatocytes, which constitutes the major cell population in the liver, should be prevented, while the apoptosis of HSCs should be promoted. We demonstrated that a low-dose of VP-16 resulted in significantly higher levels of apoptosis in LX-2 cells, and that the percentage of apoptotic and the amount of proliferation inhibition of hepatocytes were lower than those of HSCs treated with the same dosage of VP-16. Nevertheless, VP-16 is not an HSC-specific apoptosis-inducing drug for the treatment of liver fibrosis. Previous studies showed that treatment with VP-16 induced high levels of apoptosis on hepatocytes, but treatment of endotoxin-induced lethal liver injury mice with VP-16 reduced apoptosis of hepatocytes^33^,^34^. Thus, the effect of VP-16 on hepatocytes in fibrotic liver requires further investigation *in vivo*.

The production of ECM, in particular, type I collagen, is a hallmark of activated HSCs in the cirrhotic liver. Intact type I collagen promotes the persistence of activated HSCs, and ECM degradation appears to be critical for the induction of HSC apoptosis^35^. We observed that VP-16 decreased the mRNA and protein levels of type I collagen in HSCs. We also observed that VP-16 significantly decreased the mRNA and protein levels of α-SMA, which is a marker of HSCs activation. In liver tissue, MMPs and their specific inhibitors (TIMPs) play a vital role in both fibrogenesis and fibrolysis. TIMPs are profibrogenic through their inhibition of matrix degradation and promoting stellate cell survival. In activated HSCs, the expression of TIMP-1 is particularly upregulated, leading to the inhibition of MMP activity and the subsequent accumulation of matrix proteins in the extracellular space^18^. In this study, VP-16 significantly inhibited the expressions of TIMP-1 and upregulated the expression of MMP-13. Consequently, the MMP-13/TIMP-1 ratio was elevated, and VP-16 ultimately altered the balance between collagen synthesis and degradation in HSCs *in vitro*. Taken together, our data indicated that VP-16 mediates a reduction in collagen deposition both directly by reducing type I collagen protein synthesis and indirectly by increasing the MMP-13/TIMP-1 ratio, thereby potentially enhancing ECM degradation.

In summary, for the first time, our studies show that VP-16, a chemotherapeutic agent, has antiproliferative and proapoptotic effects on cultured LX-2 cells, and induces cell apoptosis through a mechanism involving the ER stress pathway and the caspase-dependent mitochondrial pathway. Moreover, we demonstrated that VP-16 inhibits collagen synthesis and induces collagen degradation in HSCs. The results deserve further investigation of VP-16 as a perspective therapeutic tool for the treatment of liver fibrosis.

## Methods

### Materials

Etoposide (VP-16, E1383), and anti-β-actin antibody (A5441) were purchased from Sigma-Aldrich (St. Louis, MO, USA). Human PDGF-BB (100-14B) was purchased from Peprotech. PD98059(S1177), Z-VAD-FMK (S7023), salubrinal (S2923) and SP600125 (S1460) were obtained from Selleck Chemical. The anti-p21 (SC-397) antibody was purchased from Santa Cruz Biotechnology (Dallas, TX, USA). The antibodies against ERK (4695P), p-ERK (4370S), cyclin B1 (4138), cdc2 (9116), PARP (9532S), cleaved PARP (5625S), caspase-7 (9491), caspase-9 (9502S), caspase-3 (9662S), cleaved caspase-3 (9661), p-PERK (3179S), eIF2a (5324P), p- eIF2a (3398P), caspase-12 (2202S), CHOP (5554), IRE1α (3294S), p-ASK1 (3764S), JNK (9258P), p-JNK (4668S), p-c-jun (3270S), Bim (2933S), Bcl-2 (2870S), Bax (5023S), and cytochrome c (11940S) and the horse-radish peroxidase-conjugated anti-mouse and anti-rabbit antibodies were obtained from Cell Signaling Technology (Danvers, MA, USA). The antibodies against XIAP (MAB822) and hPERK (AF3999) were purchased from R&D Systems (Abingdon, UK). The antibodies against type I collagen (ab138492), α-SMA (ab124964), MMP13 (ab39012), TIMP1 (ab109125) and BIP (ab21685) were obtained from Abcam (Cambridge, UK).

### Cell Culture

The human hepatic activated stellate cell line LX-2 was purchased from Merck Millipore (USA) and was cultured in Dulbecco’s modified Eagle’s medium (DMEM) supplemented with 10% fetal bovine serum in humidified air at 37 °C with 5% CO_2_ (Thermo). The normal human hepatocytes lines LO-2 and QSG-7701 were gifts from Dr. Hongli Yan of the Department of Laboratory Medicine of Changhai Hospital, Second Military Medical University, Shanghai, China. Cells were cultured in DMEM containing 10% fetal bovine serum at 37 °C in a 5% CO_2_ incubator (Thermo). VP-16 was dissolved in dimethyl sulfoxide (DMSO) at a concentration of 50 mM and stored in the dark at −20 °C. Immediately prior to the cell treatment, the VP-16 stock was diluted to 50 μM in DMEM containing 5% FBS. In all experiments, cells were seeded at the proper confluency, and 24 h later, the cells were exposed to VP-16 for the indicated time. The concentration of DMSO in culture never exceeded 1‰.

### Cell Viability Assay

Cell viability was determined using a CCK-8 kit (Dojindo, Japan). Cells were seeded into 96-well plates at 7 × 10^3^ cells/well and cultured for 24 h at 37 °C with 5% CO_2_. After treatment with 0.1% DMSO as a control or VP-16 at varying concentrations for the indicated times, 10 ul of CCK-8 solutions was added to each well of the plate. The cells were incubated for one more hour, and the absorbance of the samples (450 nm) was determined by using a scanning multiwell spectrophotometer. The cell viability was calculated using the following formula: relative cell viability (%) = (absorbance_450 nm_ of treated group − absorbance_450 nm_ of blank)/(absorbance_450 nm_ of control group − absorbance_450 nm_ of blank) × 100. The results from 3 independent experiments in triplicates are presented.

### Colony Formation Assay

LX-2 cells were seeded into six-well plates at a density of 3,000 cells/well for 24 h and then incubated with the indicated concentrations of VP-16. After 14 days of culture, the cells were washed with phosphate-buffered saline (PBS), fixed with methanol for 15 min and sequentially stained with 0.5% crystal violet for 20 min at room temperature. The crystal violet-stained colonies were photographed. The data are representative of at least three independent experiments.

### Cell Cycle Analysis

The cell cycle distribution was analyzed by flow cytometry using a Cycle TEST DNA Reagent Kit (340242, BD Biosciences) according to the manufacturer’s instructions. In brief, approximately 15 × 10^4^ cells/well were seeded in a six-well plate overnight at 37 °C; the cells were then treated with the indicated concentrations of VP-16 for 72 h. After the cell were harvested, they were immediately stained according to the instructions. The phases of the cell cycle were analyzed by flow cytometry (BD Biosciences, Aria II). The data were analyzed with a BD FACSCanto II flow cytometer (BD Biosciences, CA). All experiments were performed in triplicate and repeated three times independently.

### Annexin V/PI Analysis

The apoptosis induced by VP-16 was assessed by staining cells with an Annexin V-fluorescein isothiocyanate (FITC) Apoptosis Detection Kit (556547, BD Biosciences). Cells were treated similarly as in the cell cycle assay. The cells were collected and then washed with cold PBS twice. Then, they were resuspended in 100 μL of Annexin V binding buffer and incubated with 5 μL of FITC-conjugated Annexin V and 5 μL of propidium iodide for 15 min in the dark. Annexin V binding buffer (200 μL) was then added to each tube. Finally, the cells were examined using a BD FACSCanto II flow cytometer (BD Biosciences, CA). All experiments were performed in triplicate and repeated three times independently.

### Mitochondrial Membrane Potential (ΔΨm) Assay

The loss of ΔΨ_m_ in cells is one of the mechanisms for the induction of apoptosis, and this loss has been linked to the initiation and activation of apoptotic cascades^36^. ΔΨ_m_ was assessed by JC-1 staining (Beyotime Institute of Biotechnology, China). LX-2 cells were seeded at a density of 15 × 10^4^ cells per well in six-well plates and cultured overnight at 37 °C. Then, the cells were treated with the indicated concentrations of VP-16 for 72 h. The cells were washed with PBS and incubated with medium containing 20 μg/mL JC-1 at 37 °C for 20 min. Then, the cells were observed using a confocal fluorescent microscope. The data are representative of at least three independent experiments.

### Caspase-3 Activity Assay

Caspase-3 activity was analyzed using a caspase-3 activity assay kit (Beyotime Institute of Biotechnology, China). LX-2 cells were seeded in a 6-well culture plate and cultured overnight at 37 °C. After being treated with VP-16 as described above, cell lysates were prepared for the following experiment according to the manufacturer’s instructions.

### Western Blot Analysis

The total protein was extracted from LX-2 cells prepared with ice-cold lysis buffer (Biosharp). The protein concentrations of the cell lysates were determined with a BCA protein kit (Beyotime Institute of Biotechnology, China). Samples containing equal amounts of protein were mixed with loading buffer containing 5% 2-mercaptoethanol and then heated for 10 min at 95 °C. Twenty to thirty micrograms of protein lysates was separated on 6–12% sodium dodecyl sulfate-polyacrylamide gels and transferred to PVDF membranes (Millipore). TBST containing 5% nonfat milk was used to block nonspecific binding for 2 h at room temperature. Next, the membranes were incubated according to the instruction with the respective primary antibodies overnight at 4 °C and then treated with the appropriate HRP-conjugated secondary antibodies (1:5,000 dilutions). The signals generated by enhanced chemiluminescence (Millipore) were recorded with a CCD camera (CLINX, Shanghai, USA), according to the manufacturer’s protocols. The experiments were repeated three times.

### Quantitative Real-time PCR

The total RNA was extracted from LX-2 cells using TRIzol reagent (Invitrogen, Carlsbad, CA), and reverse transcription was carried out with 500 ng of RNA in a total reaction volume of 10 μL using PrimeScript^TM^ RT Master Mix according to the manufacturer’s instructions. Quantitative real-time PCR experiments were performed using a 500 Real-time PCR System (Applied Biosystems) using SYBR Premix Ex Taq reagents (Takara, Japan). Primers were designed and validated by Invitrogen Biotechnology Co. Ltd. All data were normalized to the expression of the human β-actin gene. The primers sequences were as follows: β-actin, F, 5′-CTGGC ACCACACCTTCTACAATG-3′, R, 5′-AATGTCACGCACGATTTCCCGC-3′; type I collagen, F, 5′-ACTGGTGAGACCTGCGTGTA-3′, R, 5′-AATCCATCGGTCATGCTCTC-3′; and α-SMA, F, 5′-GTTCCGCTCCTCTCTCCAAC-3′, R, 5′-ACGCTGGAGGACTTGCTTTT-3′.

### Statistical Analysis

The data are presented as the means ± SD of three independent experiments. For the determination of statistical significance, a one-way analysis of variance (ANOVA) and a two-way ANOVA were performed using Prism software (version 6.0, GraphPad Software, La Jolla, CA). P-values < 0.05 were considered statistically significant.

## Additional Information

**How to cite this article**: Wang, C. *et al*. Etoposide Induces Apoptosis in Activated Human Hepatic Stellate Cells via ER Stress. *Sci. Rep.* **6**, 34330; doi: 10.1038/srep34330 (2016).

## Supplementary Material

Supplementary Information
