# Supplementary material for: Etoposide Induces Apoptosis in Activated Human Hepatic Stellate Cells via ER Stress
Source: Sci Rep. 2016 Sep 29;6:34330. doi: 10.1038/srep34330 (PMC5041150; doi:10.1038/srep34330)
Supplement: Supplementary Information [file srep34330-s1.pdf]

SUPPLEMENTAL INFORMATION

Etoposide Induces Apoptosis in Activated Human Hepatic Stellate Cells via ER Stress

Chen Wang, Feng Zhang, Yu Cao, Mingming Zhang, Aixiu Wang, Mingcui Xu, Min Su, Ming Zhang, Yuzheng Zhuge

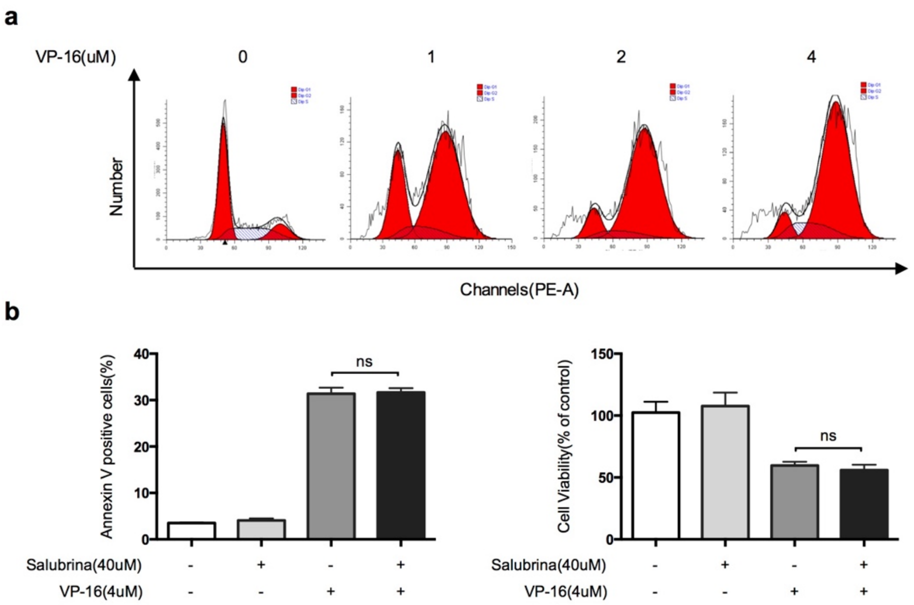

Supplemental Figure 1a. VP-16 induces G_2/M cell cycle arrest in HSCs. LX-2 cells were treated with 0-4 µM VP-16 for 72 h. Cell cycle analysis was performed using flow cytometry.

Supplemental Figure 1b. Salubrinal failed to reduce the resulting apoptosis. LX-2 cells were pretreated with Salubrinal (40 µM) for 1 h and then with 4 µM VP-16 for 72 h. Cell viability was assessed using the CCK-8 assay. Annexin V-positive cells were examined by flow cytometry. The results are expressed as the mean ± SD of three independent experiments.

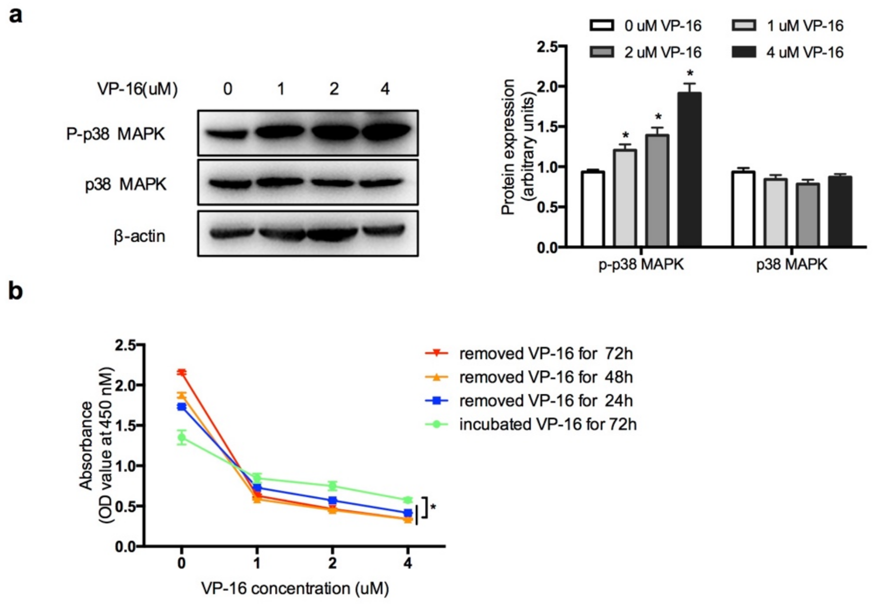

Supplemental Figure 2a. VP-16 upregulated the protein phosphorylation levels of p38 MAPK. LX-2 cells were treated with 0-4 μM VP-16 for 72 h. The protein levels of p38 MAPK and its phosphorylation were measured by western blotting. β-actin was used as a loading control. $^{*}P<0.05$ compared to control group.

Supplemental Figure 2b. The inhibitory effect remained pronounced with removal of VP-16. LX-2 cells were incubated for 72 h with VP-16 and then removed it for the indicated times. Cell viability was assessed using the CCK-8 assay. The results are expressed as the mean ± SD of three independent experiments. $^{*}P<0.05$.
